# Profiling Major Livestock Diseases in the East Gojjam Zone, Amhara Region, Ethiopia, Using Participatory Epidemiology Methods

**DOI:** 10.1002/vms3.71188

**Published:** 2026-08-24

**Authors:** Liuel Yizengaw Adnie, Haregua Yesigat, Arega Tafere, Kifle Wondimagegnehu, Dessalew Habte, Natenael Teshager, Habtamu Yalew Ayenew, Fentahun Shitaw

**Affiliations:** ^1^ Department of Veterinary Laboratory Technology College of Agriculture and Natural Resources Debre Markos University Debre Markos Ethiopia; ^2^ School of Veterinary Medicine Bahir Dar University Bahir Dar Ethiopia; ^3^ School of Veterinary Medicine Wollo University Dessie Ethiopia; ^4^ Goncha Siso Woreda Livestock and Fishery Office Motta Ethiopia

**Keywords:** amhara, East Gojjam, Ethiopia, livestock diseases, participatory epidemiology

## Abstract

**Background:**

Livestock production supports is a major source of livelihoods in East Gojjam Zone, Amhara Region, Ethiopia, but frequent infectious diseases continue to limit productivity. In areas with limited conventional surveillance, participatory epidemiology provides an effective approach for identifying priority livestock diseases based on community knowledge.

**Objectives:**

To identify and prioritize major livestock diseases affecting cattle, small ruminants and equines and to assess community knowledge regarding disease occurrence, clinical recognition, seasonality and relative impact.

**Methods:**

A cross‐sectional participatory epidemiology study involving 10 focus group discussions (FGDs) and 100 purposively selected livestock owners was conducted in five districts of East Gojjam Zone between April and December 2024. Tools included semi‐structured interviews, pairwise ranking, proportional piling, matrix scoring and seasonal calendars. Data were summarized descriptively using ranking scores and proportional piling results.

**Results:**

Blackleg, foot‐and‐mouth disease (FMD) and lumpy skin disease (LSD) were consistently ranked as the most important diseases affecting cattle. Pasteurellosis and sheep and goat pox were identified as the leading diseases of small ruminants, while African horse sickness (AHS) and strangles were prioritized amongst equines. Matrix scoring demonstrated variation amongst focus groups in recognition of disease‐specific clinical signs, reflecting differences in local knowledge and interpretation. Participants also reported frequent use of herbal remedies alongside conventional veterinary treatments, suggesting limited access to affordable animal health services.

**Conclusions:**

Priority livestock diseases remain major constraints to livestock production in East Gojjam Zone. Integrating community‐based knowledge with formal veterinary surveillance, expanding access to animal health services and strengthening farmer training may improve disease detection, reporting and control.

## Graphical abstract

Participatory epidemiology identified CBPP, LSD, FMD and sheep/goat pox as the major livestock diseases in East Gojjam, Ethiopia, highlighting strong community‐based disease recognition and emphasizing the value of integrating local knowledge into veterinary surveillance and disease control systems.

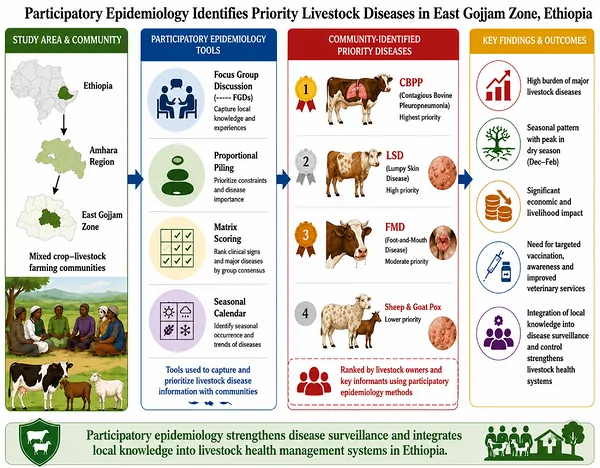

## 1 Introduction

Livestock production is widely recognized as a key component of agricultural development in many parts of sub‐Saharan Africa. Ethiopia holds the largest livestock population in Africa and ranks tenth globally. It serves as the primary source of livelihood and nutrition for over 300 million people in the region (Abate et al. 2012). In Ethiopia, as in many other developing countries, domestic animals play an essential role in both the agricultural economy and rural household welfare. They provide food products such as meat and milk, as well as non‐food services including draft power, manure for fertilization and transportation. Livestock also generate income through the sale of these products, as well as hides and skins. Beyond their economic value, livestock serve as a store of wealth and a symbol of social status within many communities (Gebrecherkos 2012; Mekuriaw and Harris‐Coble 2021).

The East Gojjam Zone in the Amhara Region is characterized by a mixed crop‐livestock farming system, in which livestock play a vital role in enhancing household resilience and sustaining livelihoods. Despite its importance, the livestock sector in the region faces numerous challenges, with endemic and emerging diseases being amongst the most pressing. Common diseases such as anthrax, blackleg, foot‐and‐mouth disease (FMD) and lumpy skin disease (LSD). Contagious bovine pleuropneumonia (CBPP) and internal and external parasites frequently affect cattle, sheep, goats and other domestic animals. These illnesses reduce productivity in meat, milk and draught power, while also increasing mortality and morbidity rates. The burden of these diseases not only compromises animal welfare but also limits household income, food availability and rural communities' ability to invest in agricultural development (Ayal et al. 2017). Furthermore, disease outbreaks often result in restrictions on livestock movement and trade, deepening the economic impact on already vulnerable households (Countryman et al. 2024; Haftu et al. 2014a).

Amongst the major livestock diseases affecting Ethiopia, anthrax, blackleg, FMD and LSD are of considerable economic and public health importance. Anthrax is caused by *Bacillus anthracis*. It remains endemic in many parts of Ethiopia and causes substantial livestock mortality. It also poses a risk of zoonotic transmission (Yadeta et al. 2020). Blackleg is caused by *Clostridium chauvoei* (Radostits et al. 1997). This highly fatal bacterial disease primarily affects young cattle and is frequently reported in mixed crop‐livestock production systems (Bahiru and Assefa 2020). FMD is one of the most economically significant transboundary animal diseases in Ethiopia. It causes production losses, reduced milk yield, decreased draft power and restrictions on livestock trade (Rasmussen et al. 2024). Similarly, LSD is a vector‐borne viral disease of cattle that has become increasingly important. Its widespread distribution has led to significant economic impacts, including decreased productivity, skin damage and mortality (Countryman et al. 2024). Recurrent outbreaks of these diseases have been reported in several regions of Ethiopia, including the Amhara Region. This underscores the need for effective surveillance and control strategies (Thompson et al. 2024).

Conventional epidemiological data in the region are limited, especially at the community level, where formal surveillance and reporting systems are often weak (Alders et al. 2020; Catley et al. 2012). In this regard, participatory epidemiology (PE) is a pertinent method that enables people to present, share and analyse their knowledge and experience to inform community‐based interventions. Unlike a questionnaire administered to an individual interviewee, which limits responses to a set number of questions, PE is participatory, empowering people to identify and find solutions to their own developmental challenges. PE has been widely applied in livestock disease surveillance and control, particularly in areas with limited veterinary services. PE combines the local knowledge of livestock owners with veterinary expertise to improve early detection, prioritisation and management of diseases. Kholik et al. (2024), conducted a study on Lombok Island, Indonesia, demonstrating that PE enabled community farmer groups to detect FMD outbreaks early and to report them to authorities promptly, thereby reducing the impact of FMD on livestock production. The study emphasized that farmers’ observations and participatory monitoring were critical for rapid disease response in resource‐limited settings (Azhar et al. 2010). Similarly, PE approaches were reported to strengthen community engagement and inform the design of locally adapted disease control interventions, particularly for endemic livestock diseases (Azhar et al. 2010; Kholik et al. 2024). These findings, along with global evidence, underscore the utility of PE as a complementary tool to conventional veterinary surveillance, improving disease detection, reporting and the planning of control strategies in mixed farming systems (Alders et al. 2020)

PE enables researchers to use indigenous knowledge to identify disease patterns, priorities and perceptions, thereby complementing conventional data sources (Alders et al. 2020; Catley et al. 2012; Muhammad et al. 2023). In the East Gojjam zone, there was no clear data or information on the priority livestock disease, which significantly affects productivity and production. Hence, this study aims to profile major livestock diseases affecting the East Gojjam Zone by using PE methods. By involving local communities in disease identification and prioritisation, the research seeks to bridge the gap between scientific surveillance and community‐level realities.

## 2 Materials and Methods

### 2.1 Study Area and Study Population

**FIGURE 1:**
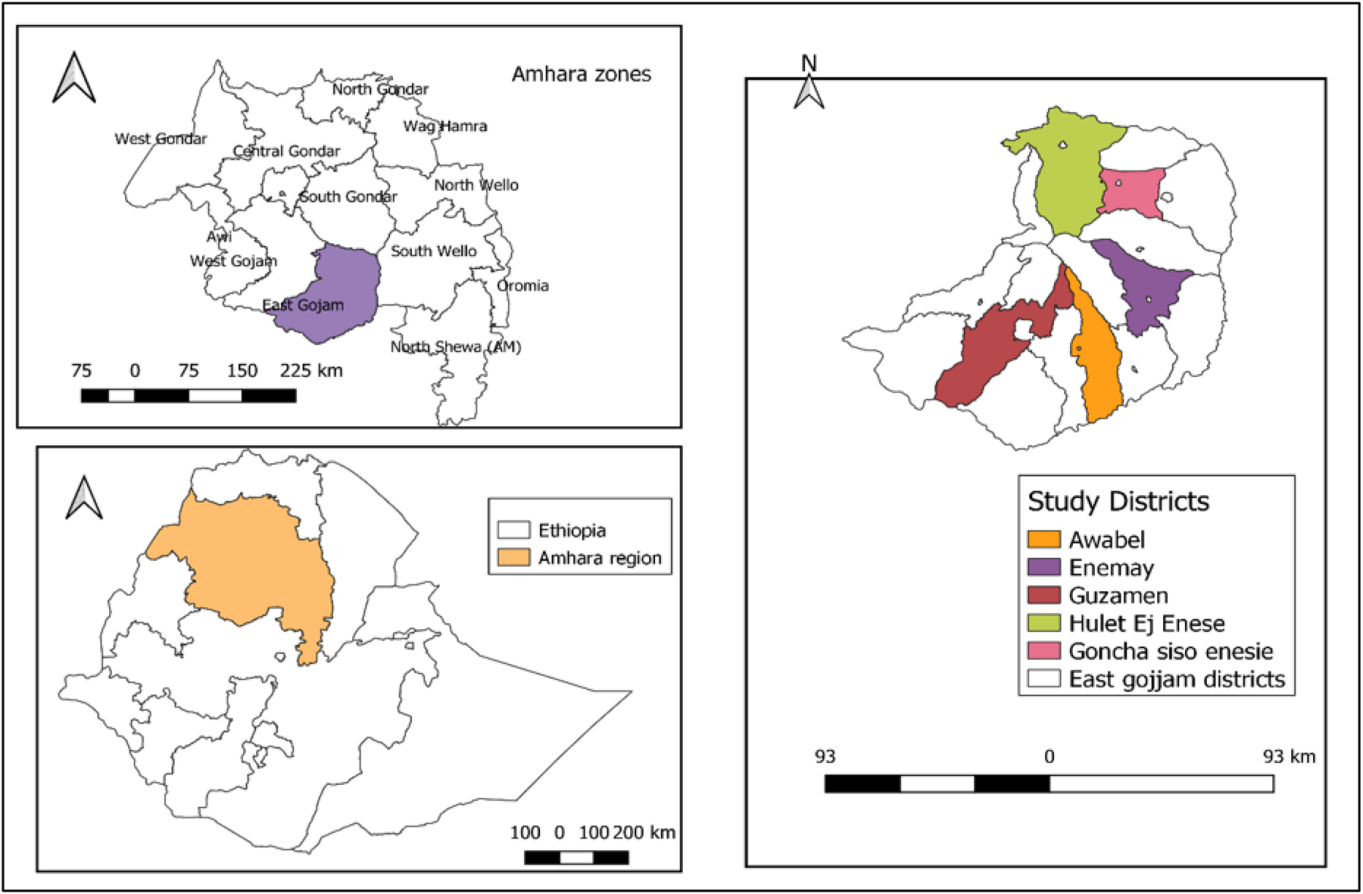
Map of the study districts of East Gojjam zone, Amhara, Ethiopia.

This study was conducted in the East Gojjam Zone, located in the Amhara National Regional State of northwestern Ethiopia between 10°33″ N and 38**°**00″ E latitude and longitude respectively with the altitude ranges from 807 to 4236 m above sea level (Average: 2060 m) (Figure 1). The zone is predominantly rural and is characterized by a high‐altitude, temperate climate that supports a mixed crop‐livestock farming system. Administratively, East Gojjam comprises several districts (woredas), each with multiple kebeles (the smallest administrative units). The area is known for its substantial livestock population, including cattle, sheep, goats, equines and poultry, which are integral to the livelihoods of local communities. Five districts, Awabel, Enemay, Gozamen, Goncha Siso Enesie and Hulet Iju Enesie, were included in the study (Figure 1; Supporting Information). The study population consisted of livestock‐owning households residing in selected woredas within the zone. These households were purposively selected based on livestock density, accessibility and the presence of veterinary services. Respondents included household heads or primary livestock caretakers who were directly involved in the day‐to‐day management of animals and thus possessed relevant knowledge, attitudes and practices related to livestock health and production.

### 2.2 Study Design, Sampling Strategy and Sample Size Determination

A cross‐sectional study using participatory epidemiological methods was carried out from April to December, 2024. A multistage sampling method was employed to select the study participants. First, the districts were purposively selected to fit a specific scientific criterion and to represent different agro‐ecological conditions and production systems. Next, *kebeles* (the smallest administrative unit) within districts, villages within *kebele* and households within villages were selected randomly. A list of kebeles was obtained from the district agricultural office, and lists of villages and households were obtained from the kebele administrative offices. Households were selected randomly using a computer‐based random number generator. Individuals within households were purposively selected, targeting those with cattle who volunteered to participate in the study. Community leaders, traditional healers, and elders with a strong bond to cattle and are known for their indigenous veterinary knowledge were chosen as key discussants (Gietaneh et al. 2023). Key informant interviews regarding the selection of group discussants and the local names for common livestock diseases were conducted at each kebele before the actual days of group discussion. One animal health expert and a traditional healer were chosen as key informants. A total of 10 kebeles, two from each district, and an average of 10 individuals per focus group discussion (FGD) were included. Based on this, a total of 100 discussants (participants) participated in this study.

### 2.3 Data Collection Procedure (PE Methods)

The PE methods used in this study were FGD, semi‐structured interviews, simple ranking and scoring, proportional piling, pairwise ranking (PWR) and matrix scoring (Barasa et al. 2008; Catley et al. 2009, Catley et al. 2013). Researchers facilitated every PE technique. Hence, equal chances were given to participants to minimize individual dominance within groups.

### 2.4 Location, Scheduling, Timing and Group Interviewing

A pilot study was conducted using formal interviews to ensure question clarity and refine the questionnaire format. Interview venues, such as schools and village halls, were selected in consultation with key informants and community opinion leaders. To accommodate participants' availability, interviews were scheduled during the dry (off) season, taking into account local seasonal activities and daily routines. All interviews were conducted in Amharic, which was universally understood by participants, eliminating the need for interpretation. FGDs included an average of 10 participants per site, with a minimum of two sessions conducted in each kebele.

### 2.5 Semi‐Structured Interview and FGD

A semi‐structured interview, guided by a checklist covering various topics, including introduction, livelihood strategies, types of livestock, challenges in livestock production, diseases, use of PE tools, access to veterinary services, existing veterinary knowledge and space for questions and comments, was employed to gather key information on past, present and future livestock‐related events. The checklist items ranged from personal introductions to concluding remarks, providing participants with an opportunity to reflect on their experiences and propose solutions for improving livestock health and productivity in their communities. Probing questions were integrated throughout the appraisal process to elicit detailed responses and ensure the consistency of the information collected (Figure S2; Supporting Information).

### 2.6 Simple and PWR

This participatory method was employed to prioritize livelihood options and assess the perceived benefits of livestock rearing in the study area. Participants were first asked to identify four primary livelihood categories. Each group received 20 stones (equivalent to five counters per item) to distribute amongst the categories based on their relative importance, following the approach outlined by Catley et al. (2009) and Ayele et al. (2016). The greater the number of stones assigned to a category, the higher its perceived importance. A similar exercise was then carried out to evaluate five key benefits of livestock rearing, with participants distributing 25 stones according to their perceived value. These exercises were conducted across 10 FGDs. The results were analysed by summing the scores for each item and averaging across all groups to determine the final ranking.

The PWR technique was used to identify the most significant livestock diseases in the study communities. The process began by listing the major diseases, which were then systematically compared in pairs, allowing participants to evaluate two diseases at a time until all possible combinations were assessed. To facilitate comprehension, diseases were presented using local terminology and illustrated with familiar objects. This activity was conducted across 10 separate FGDs. For each comparison, participants identified the disease they perceived as more impactful, and corresponding scores were assigned. The final prioritisation was based on the frequency with which each disease was favoured over others. In addition to establishing disease priorities, the method revealed key clinical signs that informed subsequent matrix scoring exercises (Alders et al. 2020; Catley et al. 2009; Mukanga et al. 2012).

### 2.7 Proportional Piling

Proportional piling was employed to rank major constraints to livestock production, evaluate disease treatment preferences and assess the relative importance and population sizes of different livestock species within the study communities. Circles were drawn on paper, each representing a specific constraint or treatment option. Participants in each focus group first identified key livestock production challenges and common treatment methods, using an approach similar to basic scoring and ranking techniques. They were then given 100 counters (stones) to distribute amongst the circles based on the perceived severity or importance of each item (Catley et al. 2012; Mukanga et al. 2012). This exercise was conducted with 10 separate focus groups, and final rankings were determined by summing the scores assigned to each item. The same method was also used to assess the relative frequency of major livestock diseases.

### 2.8 Matrix Scoring

Matrix scoring was employed to evaluate and confirm the focus groups' capacity to diagnose livestock diseases using clinical signs. In this approach, objects symbolising specific diseases were arranged along the x‐axis of a matrix, while clinical indicators were listed along the y‐axis. Participants allocated piles of stones to each disease–indicator pair, with more stones signifying a stronger perceived association. Scoring was done through group discussion and consensus. This exercise was conducted in 10 separate FGDs. The resulting scores for each disease–indicator combination were summarized using minimum, median and maximum values (Ayele et al. 2016).

### 2.9 Seasonal Calendar

The Gregorian (official) calendar was used across all surveyed communities for seasonal and temporal analyses. A horizontal timeline was created and segmented either by seasons (winter, summer, spring and autumn) or by months (September to August). Participants initially identified relative rainfall patterns by placing markers along the timeline. They were then guided to categorise the year into seasons based on distinct local characteristics. For diseases exhibiting seasonal trends, participants indicated their relative occurrence along the seasonal timeline using counters. Additionally, key risk factors and disease determinants such as rainfall, humidity and vector abundance were evaluated in relation to disease incidence. To assess the timing, prevalence, and seasonality of major livestock diseases, proportional piling was used, with participants distributing 100 beans to represent disease patterns throughout the year. The exercise included in‐depth group discussions to reach consensus, ensuring that the scores accurately captured the local realities.

### 2.10 Data Management and Statistical Analysis

A database was constructed in Microsoft Excel to store the data. The disease‐diagnosing ability of each focus group was assessed using Kendall's coefficient of concordance (W). The agreement was termed weak, moderate and good if W values were less than 0.26, between 0.26 and 0.38 (*p* < 0.05), and greater than 0.38 (*p* < 0.01–0.001), respectively. All statistical analyses were performed using STATA version 17 (Chicago, USA).

## 3 Results

### 3.1 Livelihood Categorisation

Livelihood categorisation was carried out to assess the relative importance of livestock in the study area. Using PE tools such as proportional piling, simple ranking and PWR, mixed crop‐livestock farming was identified as the primary livelihood activity. This was followed by crop production, livestock production and, lastly, trading and other activities (Table 1). The results underscore the pivotal role of integrated farming systems in supporting rural livelihoods and highlight the essential contribution of livestock within these systems.

**TABLE 1:** The major livelihood options of the community in the study districts.

Livelihood practice	Number of FGDs	Total score	Average score	Overall rank
Mixed crop‐livestock production	5 (10)	88	8.8	1
Crop production	5 (10)	66	6.6	2
Livestock production	5 (10)	30	3	3
Trading and others^a^	5 (10)	16	1.6	4

Abbreviation: FGDs, focus group discussions.

^a^Carpentry, Brocker and labouring, etc.

### 3.2 Major Constraints of Livestock Production

Shortages of feed and free grazing land, along with livestock diseases, emerged as the most critical constraints to livestock production across the study area (Figure S3; Supporting Information). However, the ranking of these challenges showed slight variation between districts, highlighting differences in local environmental conditions, resource access and the prevalence of livestock diseases.

### 3.3 Purpose of Keeping (Rearing) Livestock (Cattle, Sheep and Goats)

The five most commonly cited benefits of cattle rearing amongst participants were draught power, income generation, transportation, household consumption and socio‐cultural functions such as serving as a symbol of wealth or being used as gifts (Table 2).

**TABLE 2:** Ranking the purpose of livestock rearing in the study districts.

Purposes	Number of FGDs	Total score	Average score	Overall rank
Draught power	5 (10)	96	9.6	1
Income source	5 (10)	68	6.8	2
Consumption	5 (10)	64	6.4	3
Transportation	5 (10)	56	5.6	4
Socio‐cultural purposes	5 (10)	16	1.6	5

Abbreviation: FGDs, focus groups discussion.

### 3.4 Priority Livestock Disease (Cattle, Sheep, Goats and Equine)

#### 3.4.1 Prioritized Cattle Diseases

The major cattle diseases prioritized by participants, in descending order of importance, were blackleg, LSD, anthrax, foot and mouth disease (FMD), pasteurellosis and parasitic infections (Figure S4; Supporting Information). These priorities were established through a participatory ranking approach, in which community members identified and ranked diseases based on their perceived impact on livestock health and productivity. Blackleg, LSD and anthrax were regarded as the most critical due to their high mortality rates and the substantial economic losses incurred during outbreaks. FMD and pasteurellosis were also noted for their widespread occurrence and the significant costs associated with their treatment. Although parasitic infections were considered less fatal, they were recognized for their persistent effects on animal health, contributing to reduced growth performance and fertility over time.

#### 3.4.2 Prioritized Shoat Diseases

The major diseases affecting sheep and goats, prioritized by importance and prevalence, were pasteurellosis, sheep and goat pox, contagious ecthyma (ORF) and/peste des petits ruminants (PPR), fasciolosis and gastrointestinal parasitism (Figure S5; Supporting Information). Participants ranked these diseases based on their perceived impact on sheep and goat health and productivity. Pasteurellosis and sheep and goat pox were considered the most significant due to their high prevalence and impact on livestock mortality and productivity. ORF and PPR were also highlighted for their infectious nature and economic consequences, particularly reduced market value and productivity. Fasciolosis and GIT parasitism were recognized for their chronic effects, leading to decreased weight gain, fertility and overall health in affected animals.

#### 3.4.3 Prioritized Equine Diseases

The major diseases affecting equines, ranked by importance and prevalence, were wounds, African horse sickness (AHS), strangles, anthrax, gastrointestinal parasitism and epizootic lymphangitis (Figure S6; Supporting Information). Participants identified these diseases as the most critical threats to equine health, with wounds and AHS being considered the most significant due to their potential for high widespread occurrence, impact on performance and high mortality rate. Strangles and anthrax were also highlighted for their widespread occurrence and their economic consequences, particularly in terms of treatment costs and animal losses. GIT parasitism and epizootic lymphangitis, while less fatal, were acknowledged for their chronic effects on equine health, leading to reduced productivity and increased veterinary care requirements.

### 3.5 Matrix Scoring Results

The matrix scores for common cattle, shoat, and equine diseases, along with their associated clinical indicators, are summarized in Tables 3, 4, 5, respectively. Weak to moderate agreement (*W* = −0.55 to 0.58; *p* = 0.001) was observed across the focus groups for all diseases and their respective clinical indicators.

**TABLE 3:** Matrix score of major cattle diseases and clinical signs in 10 different focus groups.

		Median score (range)					
Clinical sign	Kendall (*W*)	Black leg	LSD	Anthrax	FMD	Pasteurellosis	Parasitism
Coughing	0.5793***	0.4 (0–2)	2 (1–3)	1.6 (1–2)	2.8 (1–6)	18 (16–19)	5.2 (4–9)
Crepitation	−0.4299***	20.4 (12–25)	3.6 (2–7)	3.4 (1–6)	1.2 (0–3)	0.2 (0–1)	1.2 (0–3)
Diarrhea	0.3402**	0.2 (0–1)	1.6 (0–4)	3 (1–6)	1.6 (0–3)	5.8 (3–8)	17.8 (13–23)
Emaciation	0.4828***	0.8 (0–3)	3.4 (2–6)	1.6 (0–5)	3.6 (2–6)	4.8 (4–6)	15.8 (10–20)
Enlarged lymph node	−0.4184**	7.2 (6–9)	7 (5–9)	6.2 (5–8)	5 (3–8)	2.6 (0–5)	2 (1–3)
Fever/dryness	−0.4828***	7.6 (6–8)	5.2 (3–6)	7.2 (6–8)	4.4 (3–6)	4 (2–6)	1.6 (0–3)
High mortality	−0.4575***	7 (5–9)	2.8 (2–4)	11.6 (10–15)	2.8 (1–5)	4 (1–6)	1.8 (0–4)
Lameness	−0.3080*	7 (3–9)	6 (3–8)	2.6 (2–4)	12.6 (9–16)	0.6 (0–2)	1.2 (0–3)
Nasal discharge	0.4874***	0.4 (0–2)	3 (1–5)	1.6 (0–3)	5.6 (3–8)	16 (14–18)	3.4 (3–5)
Salivation	0.2552*	1.2 (0–2)	3.2 (2–6)	2 (1–5)	18.6 (14–22)	2.8 (1–5)	2.2 (1–3)
Skin swelling	−0.3011*	4.8 (3–8)	12.2 (6–19)	4.6 (2–8)	3.2 (1–5)	0.6 (0–3)	4.6 (3–6)
Vesicle on feet and mouth	0.4138**	0.6 (0–2)	3.8 (2–6)	2 (0–6)	20.8 (15–25)	0 (0)	2.8 (0–6)

*Note: W* Kendall's coefficient of concordance for median (*W* < 0.26 = weak, 0.26< *W* < 0.38 = moderate, *W* > 0.38 = good) *p < 0.05, **p < 0.01, ***p < 0.001.

**TABLE 4:** Matrix score of major shoat diseases and clinical signs in 10 different focus groups.

		Median score (range)				
Clinical sign	Kendall (*W*)	Pasteurellosis	Sheep and goat pox	ORF and PPR	Fasciolosis	GIT parasitism
Bottle neck	−0.5467**	1.8 (0–3)	0.8 (0–2)	0.4 (0–1)	19.2 (18–20)	7.8 (7–9)
Coughing	−0.1800	11.2 (9–13)	0.6 (0–1)	3.6 (3–4)	5.4 (4–6)	9.2 (8–11)
Diarrhea	−0.3200*	2.2 (2–3)	1.2 (1–2)	14.2 (11–15)	5.2 (5–6)	7.2 (7–8)
Fever/dryness	0.3533*	6.8 (6–8)	8 (4–10)	12 (10–18)	2 (2)	1.2 (0–3)
High mortality	0.0300	8.6 (7–10)	3.8 (3–5)	10.8 (10–12)	5.8 (3–7)	1 (0–2)
Mouth and tongue sore	0.2233	1.4 (0–2)	6.4 (5–7)	19.8 (18–24)	1.6 (0–2)	0.8 (0–1)
Nasal discharge	0.2200	7.2 (6–9)	4.6 (3–5)	11.2 (9–12)	2.8 (2–3)	4.2 (2–6)
Rough Hair coat	−0.2833*	0.8 (0–1)	6 (4–8)	3.2 (1–6)	7 (5–9)	13 (11–15)
Salivation	0.3200*	5.4 (3–9)	7.8 (6–12)	12.8 (10–14)	2.8 (1–4)	1.2 (1–2)
Skin swelling	0.3800*	0 (0)	20.8 (19–25)	8.2 (5–10)	0.4 (0–2)	0.6 (0–2)
Wound on foot	0.1900	0.4 (0–2)	7.6 (6–10)	20 (15–22)	1.4 (0–5)	0.6 (0–2)

*Note: W* Kendall's coefficient of concordance for median (*W* < 0.26 = weak, 0.26 < *W* < 0.38 = moderate, *W* > 0.38 = good) *p < 0.05, **p < 0.

**TABLE 5:** Matrix score of major equine diseases and clinical signs in 10 different focus groups.

		Median score (range)					
Clinical sign	Kendall (*W*)	Wound	AHS	Strangle	Anthrax	GIT parasitism	Epizootic lymphangitis
Back sore	0.4346*	20 (18–22)	0.4 (0–1)	3.4 (3–4)	0 (0)	0.2 (0–1)	6 (5–7)
Belly swelling	0.1333	2.8 (2–3)	2.2 (2–3)	8.8 (7–10)	7.6 (7–10)	5 (5)	3.6 (3–4)
Colic	−0.1402	0.2 (0–1)	5 (5)	5.8 (5–8)	6.2 (6–7)	9.2 (8–10)	3.6 (1–5)
Coughing	−0.2069	0.8 (0–2)	5 (5)	18 (15–20)	3 (3)	2.4 (2–3)	0.8 (0–2)
Emaciation	0.2529*	4.2 (4–5)	3 (3)	3.4 (3–5)	2.8 (2–4)	11.8 (11–12)	4.8 (4–6)
Esophageal sore and abscess	0.5172***	4.8 (4–5)	0.2 (0–1)	16.2 (16–17)	2 (2)	2 (2)	4.8 (4–5)
Lameness	0.2322	8.8 (6–10)	2.4 (1–4)	3 (3)	4.4 (4–6)	2 (2)	9.4 (9–10)
Nasal discharge	−0.0161	3 (3)	7 (7)	8.6 (8–9)	3 (3)	4.8 (4–5)	3.6 (3–5)
Nostril dilation	−0.3149*	1.2 (0–2)	13.4 (10–16)	6 (6)	3.2 (3–4)	4.2 (3–6)	2 (1–3)
Respiratory distress	−0.1563	0.8 (0–2)	13.4 (9–18)	10.4 (10–13)	1.2 (0–2)	2.2 (2–3)	2 (1–2)
Sudden death	−0.3931*	1.2 (1–2)	10 (8–12)	4.2 (4–5)	12.2 (9–12)	1 (1)	1.4 (0–2)

*Note: W* Kendall's coefficient of concordance for median (*W* < 0.26 = weak, 0.26 < *W* < 0.38 = moderate, *W* > 0.38 = good) *p < 0.05, ***p < 0.001.

### 3.6 Seasonal Variation in Livestock Disease Occurrence (Cattle, Sheep and Goats)

Overall, the highest incidence of livestock diseases was reported in winter, followed by autumn, summer and spring. However, variations in disease occurrence were observed across the different districts, as shown in Figure S7, Supporting Information. These differences suggest that local climatic conditions, environmental factors and livestock management practices may significantly influence the seasonal patterns of disease prevalence.

### 3.7 Livestock Disease Treatment Options of the Community

The majority of the discussants indicated that modern veterinary drugs were their primary choice for treating livestock diseases, followed by traditional herbal remedies (Table 6). However, a significant proportion of respondents reported using both modern and traditional treatments in combination. In addition to these common treatment methods, some discussants noted that some herders do not seek any form of treatment when their animals fall ill. Instead, these herders relied on prayer, believing it would cure their sick animals. This variation in treatment practices highlights the diverse approaches within the community and the complex factors influencing disease management. While modern veterinary services are preferred for their effectiveness, traditional remedies and belief‐based practices continue to play a role in livestock health management. The coexistence of these treatment methods underscores the importance of understanding local practices when designing interventions to improve livestock health and productivity.

**TABLE 6:** Percentage of disease treatment options in 5 different focus groups.

Disease treatment preference	Name of districts						
	Enemay	Gozamen	Hulet iju	Goncha	Awabel	Range	Overall score
	No. of FGs = 2	No. of FGs = 2	No. of FGs = 2	No. of FGs = 2	No. of FGs = 2		
Modern (veterinary)	55%	50%	40%	45%	55%	40–55	49%
Traditional (herbal)	25%	25%	35%	30%	25%	25–35	28%
Both	15%	20%	15%	15%	15%	15–20	16%
Nothing	5%	5%	10%	10%	5%	5–10	7%
Overall score	100%	100%	100%	100%	100	5%–55%	100%

## 4 Discussion

Livestock possess considerable economic and social importance in the East Gojjam Zone. This study highlights the vital role that livestock plays in supporting the livelihoods of smallholder farmers in the region. However, despite their significance, livestock productivity is substantially constrained by major challenges, particularly the widespread occurrence of various diseases (Yizengaw et al. 2026). These constraints are consistent with findings from similar studies in Ethiopia and other countries, highlighting the need for focused interventions to address these issues (Gizaw et al. 2020). This study identified livestock diseases as the primary constraints affecting livestock production, productivity and the livelihoods of smallholder farmers. These findings align with previous studies by Abate et al. (2012) and Ayele et al. (2016), as well as the unpublished report from the East Gojjam Zone Agriculture Development Office, all of which highlight similar challenges. The use of indigenous knowledge to identify major livestock‐related problems enabled community members to actively share, discuss and analyse their experiences and practices (Gizaw et al. 2020). Furthermore, this study confirmed that participatory methods and approaches are exploratory, empowering and innovative. They enable both communities and field researchers to investigate specific livestock challenges collaboratively, understand local disease characterisations, and identify context‐appropriate solutions. This observation is consistent with the findings of Ayele et al. (2016), Catley et al. (2012) and Gizaw et al. (2020).

In the present study, participants identified mixed farming as the primary livelihood strategy, followed by crop production and livestock rearing as individual components. This finding aligns with the report by Ayele et al. (2016), who noted that mixed farming is the predominant livelihood system amongst communities in the Kembata Zone of southern Ethiopia. Within this system, livestock serve as a secondary means of livelihood, following crop production (Tegegne et al. 2013). For example, studies conducted in the Sinana District (Bale Zone) and Diga District (Western Ethiopia) reported that livestock contributed 25%–41% and 33%–36% to household livelihoods, respectively (Abate et al. 2012; Duressa et al. 2014). In contrast, a study in Dandi District, central Ethiopia, found that only 2.6% of respondents considered livestock to be their primary source of livelihood (Duguma et al. 2012).

Farmers in East Gojjam keep livestock as an integral part of sustaining their household livelihoods. This study confirmed the substantial contribution of livestock to rural well‐being. However, livestock productivity is hindered by several constraints, with shortages of feed and grazing land, as well as diseases, being the most significant. These findings are consistent with previous studies by Moges and Bogale (2012) in Lay Armacheho District, Northwestern Ethiopia, and Ayele et al. (2016) in Kembata Tambaro Zone, Southern Ethiopia, both of which identified feed scarcity, limited grazing land and disease as major constraints. Participants in this study also highlighted reproductive challenges, including limited access to artificial insemination services, the unavailability of selected breeding bulls and inadequate veterinary services. Similar concerns regarding veterinary service limitations have been reported in Ethiopia (Ayele et al. 2016; Baheriw et al. 2013; Moges and Bogale 2012) and in Nigeria, where service provision was found to be insufficient (Ghali‐Mohammed et al. 2025; Ibrahim 2014).

Study participants prioritized blackleg, LSD, anthrax, FMD, pasteurellosis and parasitic infections as the most prevalent diseases affecting cattle. These findings are consistent with reports from the Ethiopian Ministry of Agriculture, which also documented the frequent occurrence of these diseases in various regions. Moges and Bogale (2012) similarly identified anthrax, LSD and blackleg as major livestock diseases in Lay Armacheho District. Other studies have reported blackleg, mastitis, LSD, FMD, ringworm, Pneumonia and fasciolosis as common diseases in the vicinity of Haramaya Town, Ethiopia (Neja and Gari 2020). Interestingly, the disease prioritisation observed in this study contrasts with findings from the South Omo Zone in Southwestern Ethiopia, where communities ranked Septicaemic pasteurellosis, Trypanosomiasis, blackleg, CBPP, and FMD as the most significant (Molla and Delil 2015). Similarly, another study identified different priority diseases such as mastitis, blackleg, Pneumonia, heartwater, FMD, anthrax and various parasitic infections (Ayele et al. 2016). These variations are likely attributable to differences in agroecological conditions, which significantly influence the epidemiology and distribution of livestock diseases across regions.

The predominance of blackleg, LSD, anthrax, FMD, pasteurellosis and parasitic infections in East Gojjam may be influenced by agroecological conditions, livestock management and biosecurity measures. Specifically, blackleg outbreaks are favoured by soil contamination with *Clostridium chauvoei* spores and often occur in areas with seasonal rainfall and intensive grazing (Tres et al. 2025). In contrast, anthrax persists due to the long‐term survival of spores in alkaline soils, while flooding events can expose contaminated grazing areas and trigger new outbreaks. Regarding LSD, frequent occurrences are attributed to vectors such as mosquitoes (*Aedes* spp., *Culex* spp.), biting flies (*Stomoxys calcitrans*) and ticks (Issimov et al. 2020). Periods of high rainfall and humidity in East Gojjam further increase vector populations and disease transmission (Ayal et al. 2017). For FMD, communal grazing and livestock markets facilitate its spread and unrestricted movement and inadequate quarantine practices also contribute to its spread (Aman et al. 2020; Seyoum and Tora 2023). Similarly, pasteurellosis outbreaks are driven by stressors, including feed shortages, transportation, abrupt climate changes and overcrowding (Godde et al. 2021). Finally, gastrointestinal parasitic infections are linked to moisture, contaminated grazing lands and poor deworming (Terfa et al. 2023). Notably, the limited use of biosecurity measures, such as quarantining new animals, vaccination, vector control and proper carcass disposal, may further increase disease transmission within and between herds (Gizaw et al. 2020; Yizengaw et al. 2026).

In this study, blackleg and LSD were ranked by livestock owners as the most important diseases affecting their animals. This prioritisation was primarily attributed to the significant economic losses associated with high mortality rates and the visibly severe clinical manifestations of these diseases. Respondents emphasized the sudden onset and fatality of blackleg, particularly in young cattle, as well as the disfiguring skin nodules and reduced market value resulting from LSD infections (Mulugeta et al. 2025). Beyond disease ranking, participants demonstrated a sound understanding of the clinical symptoms associated with both conditions. Their descriptions, including swelling and lameness for blackleg and nodular skin lesions and fever for LSD, closely mirrored the clinical presentations outlined in standard veterinary references (Radostits et al. 1997). This alignment suggests a high level of experiential knowledge amongst livestock keepers, likely derived from repeated exposure to these diseases and their impacts on herd health and productivity (Gizaw et al. 2020).

In small ruminants, discussants identified pasteurellosis, sheep and goat pox and Orf as the most significant diseases. This finding is consistent with the results of Moges and Bogale (2012) and Haftu et al. (2014a). However, it differs slightly from the findings of G. Abraha (2007) and N. H. Abraha (2007), who reported Coenuruses as the most prevalent disease affecting sheep and goats in the Atsbi Wonberta area. This variation may be attributed to differences in the agroecological conditions of the study areas, which can influence the epidemiology and distribution of diseases.

Participants identified wounds and AHS as the most pressing health issues affecting equines. These problems were largely linked to poor management practices, including inadequate maintenance of harnessing equipment used during draft work, limited availability of veterinary services and an overall lack of proper care and attention towards equine health within the community. These findings align with reports from the zonal livestock office and echo similar observations documented in other regions of Ethiopia (Ayele et al. 2016; Duguma et al. 2012; Haftu et al. 2014a). A comparable situation has been noted in Nigeria, where Waziri and Yunusa (2014) found that only 17% of surveyed communities had access to veterinary and extension services, highlighting widespread challenges in equine healthcare (Oladele 2004).

The clinical presentations of livestock diseases described by our discussants were consistent with reports from various regions of Ethiopia, including pastoral cattle in Afar (Shiferaw et al. 2010), Somali (Molla et al. 2013), Oromia (Rufael et al. 2008), Amhara (Moges and Bogale 2012), Tigray (Haftu et al. 2014a; Haftu et al. 2014b), and the Southern Nations, Nationalities and Peoples' Region (SNNPR) (Ayele et al. 2016). These findings suggest that while there is some consistency in identifying clinical signs, there is also noticeable variability in how different groups perceive and interpret these indicators. This finding is inconsistent with previous reports from various regions of Ethiopia (Ayele et al. 2016; Eshetu 2003; Haftu et al. 2014a; Haftu et al. 2014b; Molla et al. 2013; Rufael et al. 2008; Tadesse 2003). Livestock owners traditionally describe livestock diseases by their local names, and some diseases are locally known, even though communities in different regions speak different languages. For instance, blackleg (Gangraena emphysematosa caused by *Clostridium chauvoei*) is known as Aba Gorba, and bovine pasteurellosis as Gororsa in this study area. Local veterinarians are well acquainted with local disease nomenclature, which helps translate farmers' perceptions into modern veterinary terminology (Gizaw et al. 2020). The negative values in the matrix scoring indicate a weak to moderate level of disagreement or inconsistency in how the groups identified and ranked the clinical signs, suggesting varying interpretations and perceptions of disease indicators across groups.

Moreover, the incidence of livestock diseases was notably higher during the winter months (December to February), a trend that aligns with findings reported in the veterinary literature (Ayal et al. 2017; Radostits et al. 1997). This seasonal spike in disease occurrence may be attributed to a combination of environmental and management‐related stressors prevalent during the dry season. During this period, the scarcity of water and forage leads to nutritional deficiencies that compromise the animals' immune systems and increase their susceptibility to infections (Ayal et al. 2017; Desta 2022; Radostits et al. 1997). In East Gojjam Zone, diseases such as pasteurellosis, FMD and internal parasitism were reported more frequently during the winter season. The dry and dusty conditions may predispose animals to respiratory illnesses like pasteurellosis, while weakened immunity and increased movement of animals in search of feed and water facilitate the spread of FMD (Marru et al. 2013; Radostits et al. 1997). Internal parasites, though typically associated with wetter conditions, may persist due to poor pasture management and close animal contact at limited water points (Waret‐Szkuta et al. 2011). Inadequate housing and limited access to veterinary services during this season further exacerbate disease risks (Yizengaw et al. 2026). Therefore, the winter months represent a critical period that requires targeted interventions such as strategic vaccination, supplemental feeding and improved access to veterinary care to reduce the burden of seasonal disease outbreaks and protect livestock productivity (Bett et al. 2017). For disease control, livestock owners in East Gojjam use a combination of modern veterinary drugs and traditional herbal remedies. The reliance on herbal remedies was particularly pronounced due to limited access to veterinary services and the high cost of modern drugs (Abebe et al. 2022). This practice reflects the broader trend across Ethiopia, where many livestock owners rely on traditional remedies as a primary means of animal health management (Ayele et al. 2016; Duguma et al. 2012; Feyera et al. 2017; Teshale Sori et al. 2004).

## 5 Conclusion

This study highlights the crucial role livestock play in sustaining the livelihoods of smallholder farmers in the East Gojjam Zone, Ethiopia, while also revealing significant productivity challenges due to the widespread prevalence of diseases. Consistent with findings from similar research in Ethiopia and other countries, these constraints underscore the urgent need for targeted interventions. PE and community‐based prioritisation methods proved effective in incorporating indigenous knowledge and local perceptions, thereby enhancing the relevance and sustainability of disease control efforts. Livestock owners identified blackleg, LSD, FMD, pasteurellosis and fasciolosis as the most pressing health concerns, providing a locally grounded basis for focused control programmes and resource allocation. However, variability in the recognition of clinical signs, as revealed through matrix scoring exercises, indicates a need for further training and standardisation in disease diagnosis. The coexistence of traditional herbal remedies with modern veterinary treatments also reflects challenges in accessing affordable and reliable veterinary services. Overall, the findings emphasize the importance of improving veterinary access and strengthening livestock management practices to mitigate disease impacts and enhance productivity in the region.

### 5.1 Limitations of the Study

While this study provides valuable insights into livestock health and production challenges in East Gojjam Zone through PE methods, several limitations should be acknowledged. First, the participatory tools used—such as proportional piling, matrix scoring and PWR—are inherently subjective, relying heavily on participants' perceptions, experiences and recall ability. This introduces the possibility of recall bias or inconsistencies in disease recognition, especially where clinical signs of different diseases overlap. Second, the study's reliance on FGDs may have limited the diversity of opinions, as dominant individuals within groups may have influenced outcomes, and marginalized voices (such as women or youth) may not have been adequately represented, despite efforts to ensure inclusivity.

Third, although the study was conducted in multiple districts, the geographic scope remains limited to East Gojjam Zone, and findings may not be generalisable to other regions with different agroecological, socio‐economic and disease profiles. Fourth, the diagnosis of livestock diseases was based solely on community knowledge and observable clinical signs, without laboratory confirmation. This limits the ability to validate disease prioritisation with scientific rigour, potentially undermining the reliability of the findings. Fifth, logistical and resource constraints prevented follow‐up assessments or the inclusion of seasonal variability through longitudinal data collection, which would have enhanced the robustness of the temporal analysis.

Finally, while the study highlights the use of traditional remedies and limited access to veterinary services, it does not deeply explore the effectiveness, safety, or pharmacological basis of these practices. This leaves an important gap in understanding how traditional and modern veterinary systems interact and could be better integrated. Despite these limitations, the participatory approach provided rich, context‐specific insights that can inform more locally appropriate and sustainable livestock health interventions.

## Author Contributions

**Liuel Yizengaw Adnie**: designed and performed the study, analysed and interpreted the data, wrote the paper. **Haregua Yesigat**: conceived and designed the study, analysed and interpreted the data, wrote the paper. **Kifle Wondimagegnehu**: conceived and designed the study, analysed and interpreted the data, wrote the paper. **Arega Tafere**: conceived and designed the study, analysed and interpreted the data, wrote the paper. **Dessalew Habtie**: conceived and designed the study, analysed and interpreted the data, wrote the paper. **Habtamu Yalew Ayenew**: conceived and designed the study. **Natenael Teshager**: conceived and designed the study. **Fentahun Shitaw**: collected the data. All the authors participated in the approval of the final manuscript.

## Funding

This study was supported by Debre Markos University, College of Agricultural and Natural Resources.

## Ethics Statement

Ethical approval for this study was obtained from the Research Ethics Review Committee of DMU, Ethical Clearance Committee ref. no. 876/10/2024, Debre Markos University, Ethiopia. Before data collection, permission was obtained from East Gojjam Zone Agricultural Offices and district livestock authorities. All participants were informed about the objectives of the study, the voluntary nature of participation, and the confidentiality of the information provided. Verbal informed consent was obtained from each participant before conducting FGDs, semi‐structured interviews and participatory epidemiological exercises. Participants were assured that their responses would be used solely for research purposes and reported anonymously. All the methods were carried out in accordance with relevant guidelines and regulations.

## Conflicts of Interest

The authors declare no conflicts of interest.

## Supporting information




**Supporting file 1**: vms371188‐sup‐0001‐SuppMat.docx

## Data Availability

The datasets during and/or analysed during the current study are available from the corresponding author upon reasonable request.
