# Supplementary material for: Profiling Major Livestock Diseases in the East Gojjam Zone, Amhara Region, Ethiopia, Using Participatory Epidemiology Methods
Source: Vet Med Sci. 2026 Aug 24;12(5):e71188. doi: 10.1002/vms3.71188 (PMC13504011; doi:10.1002/vms3.71188)
Supplement: Supplementary file 1 [file VMS3-12-e71188-s001.docx]

Figure 1: Map of the study districts of East Gojjam zone, Amhara, Ethiopia

Figure 2: Semi-structured interview and focus group discussion at the districts

Proportion

| Name of districts | Feed shortage | Disease | Reproduction problem | Veterinary service | Capital |
| --- | --- | --- | --- | --- | --- |
| Enemay | 11 | 10 | 4 | 3 | 2 |
| Gozamen | 10 | 11 | 3 | 4 | 2 |
| Hulet Iju | 10 | 8 | 7 | 3 | 2 |
| Goncha Siso | 9 | 10 | 6 | 3 | 2 |
| Awabel | 10 | 9 | 6 | 3 | 2 |

Figure 3. Major constraints of livestock production in the study districts.

Proporton

| Name of disease | Enemay | Gozamen | Hulet Iju | Goncha Siso | Awabel | Total |
| --- | --- | --- | --- | --- | --- | --- |
| Anthrax | 7 | 8 | 0 | 8 | 3 | 26 |
| Black leg | 8 | 7 | 3 | 7 | 6 | 31 |
| Endo and external parasitisim | 0 | 0 | 6 | 0 | 8 | 14 |
| Fasciolosis | 6 | 3 | 0 | 0 | 0 | 9 |
| FMD | 0 | 6 | 7 | 3 | 6 | 22 |
| LSD | 3 | 6 | 8 | 6 | 7 | 30 |
| Pasteruolosis | 6 | 0 | 6 | 6 | 0 | 18 |

Figure 4: Major cattle diseases in the study districts.

Note: FMD: foot-and-mouth disease; LSD: lumpy skin disease.

Proportion

| Name of disease | Enemay | Gozamen | Hulet Iju | Goncha Siso | Awabel | Total |
| --- | --- | --- | --- | --- | --- | --- |
| Anthrax | 0 | 0 | 7 | 1 | 1 | 9 |
| CCPP/pnumonia | 0 | 2 | 0 | 0 | 0 | 2 |
| Fasciolosis | 6 | 7 | 3 | 4 | 1 | 21 |
| GIT parasitisim | 5 | 0 | 4 | 7 | 2 | 18 |
| ORF/PPR | 5 | 9 | 5 | 0 | 7 | 26 |
| Pasteruolosis | 7 | 7 | 2 | 8 | 7 | 31 |
| Septicemia | 0 | 0 | 0 | 5 | 0 | 5 |
| Sheep and goat pox | 4 | 5 | 9 | 5 | 7 | 30 |
| Foot root | 3 | 0 | 0 | 0 | 5 | 8 |

Figure 5: Major shoat diseases in the study districts

Note: CCPP: contagious caprine pleuropneumonia; GIT, gastrointestinal tract; ORF/PPR: orf/peste des petits ruminants.

Proportion

| Name of disease | Enemay | Gozamen | Hulet Iju | Goncha Siso | Awabel | Total |
| --- | --- | --- | --- | --- | --- | --- |
| AHS | 6 | 6 | 7 | 6 | 3 | 28 |
| Anthrax | 4 | 3 | 6 | 7 | 4 | 24 |
| Epizootic lymphagities | 0 | 6 | 0 | 0 | 4 | 10 |
| GIT parasitism | 0 | 1 | 6 | 6 | 6 | 19 |
| Lung worm | 3 | 0 | 2 | 0 | 2 | 7 |
| Strangle | 6 | 4 | 4 | 4 | 6 | 24 |
| Tetanus | 0 | 3 | 2 | 3 | 0 | 8 |
| Wound | 11 | 7 | 3 | 4 | 5 | 30 |

Figure 6. Major equine disease in the study districts

Note**:** HS, African horse sickness; GIT, gastrointestinal tract

Proportion (No)

| Name of Districts | Automen(September-November) | Spring(March -May) | Winter(December-February) | Summer(June-August) |
| --- | --- | --- | --- | --- |
| Enemay | 7 | 6 | 4 | 3 |
| Gozamen | 4 | 3 | 6 | 7 |
| Hulet Iju | 3 | 4 | 7 | 6 |
| Goncha Siso | 6 | 3 | 7 | 4 |
| Awabel | 6 | 4 | 7 | 3 |
| Total | 26 | 20 | 31 | 23 |

Figure 7: Seasonal variations of livestock disease (cattle, sheep, and goats)
